# Relationship of Success Rate for Balloon Adhesiolysis with Clinical Outcomes in Chronic Intractable Lumbar Radicular Pain: A Multicenter Prospective Study

**DOI:** 10.3390/jcm8050606

**Published:** 2019-05-03

**Authors:** Jun-Young Park, Gyu Yeul Ji, Sang Won Lee, Jin Kyu Park, Dongwon Ha, Youngmok Park, Seong-Sik Cho, Sang Ho Moon, Jin-Woo Shin, Dong Joon Kim, Dong Ah Shin, Seong-Soo Choi

**Affiliations:** 1 Department of Anesthesiology and Pain Medicine, Asan Medical Center, University of Ulsan College of Medicine, Seoul 05505, Korea; anesthesia.pains@gmail.com (J.-Y.P.); sjinwoo@hotmail.com (J.-W.S.); gochokdj@naver.com (D.J.K.); 2 Department of Neurosurgery, Spine and Joint Research Institute, Guro Cham Teun Teun Hospital, Seoul 08392, Korea; jivago91@daum.net; 3 Department of Neurosurgery, Yonsei Barun Hospital, Seoul 07013, Korea; goodi06@hanmail.net (S.W.L.); drhdw722@gmail.com (D.H.); yungmok76@hanmail.net (Y.P.); 4 Department of Neurosurgery, Himchan Hospital, Bupyeong 21399, Korea; foryou94@hanmail.net; 5 Department of Occupational and Environmental Medicine, College of Medicine, Dong-A University, Busan 49201, Korea; 0361pt@hanmail.net; 6 Department of Orthopedic Surgery, Seoul Sacred Heart General Hospital, Seoul 02488, Korea; msh124@paran.com; 7 Department of Neurosurgery, Spine and Spinal Cord Research Institute, Severance Hospital, Yonsei University College of Medicine, Seoul 03722, Korea

**Keywords:** low back pain, lumbar radicular pain, lumbar spinal stenosis, balloon decompression, ZiNeu catheter, epidural adhesiolysis

## Abstract

Combined balloon decompression and epidural adhesiolysis has been reported to be effective in refractory lumbar spinal stenosis. Many cases of intractable stenosis have symptom-related multiple target sites for interventional treatment. In this situation it may not be possible to perform balloon adhesiolysis, or even only epidural adhesiolysis, for all target sites. Therefore, this multicenter prospective observational study aimed to evaluate the relationship of successful ballooning rate for multiple target sites with clinical outcome. Based on the ballooning success rate of multiple target sites, the patients were divided into three groups: below 50%, 50–85%, and above 85% ballooning. A greater ballooning success rate for multiple target sites provided a more decreased pain intensity and improved functional status in patients with chronic refractory lumbar spinal stenosis, and the improvement was maintained for 6 months. The estimated proportions of successful responders according to a multidimensional approach in the below 50%, 50–85%, and above 85% balloon success groups at 6 months after the procedure were 0.292, 0.468, and 0.507, respectively (*p* = 0.038). Our study suggests the more successful balloon adhesiolysis procedures for multiple target lesions are performed, the better clinical outcome can be expected at least 6 months after treatment.

## 1. Introduction

Low back pain and/or radiating leg pain is a common medical and social condition in the general population [1,2,3]. Various causes, such as muscle sprain, lumbar facet joint syndrome, herniated intervertebral disc disease, and spinal stenosis, contribute to the development of lower back pain and/or leg pain [1]. Lumbar spinal stenosis is one of the most common spinal diseases in elderly patients [3]. Over approximately 30% of elderly patients complain of lower back pain or leg pain, and symptomatic spinal stenosis is present in approximately 1.7–8.4% of the population [2,4]. Lumbar stenosis is also associated with functional disabilities such as neurogenic claudication [5,6]. 

The initial management of lumbar radicular pain and/or low back pain generally involves conservative treatment such as physiotherapy, medication, and epidural injections [7,8]. Epidural adhesion induced by degenerative spinal stenosis, previous spine surgery, or intervertebral disc herniation is known to be associated with a poor effectiveness of epidural injections [8,9]. In cases of the poor effectiveness of fluoroscopically guided epidural injections, it is recommended to try percutaneous epidural adhesiolysis first rather than surgery [8,10,11]. Percutaneous epidural adhesiolysis has been commonly performed with various specifically designed catheters [8,11,12,13]. However, incomplete or poor effectiveness are not uncommon in patients with severe spinal stenosis or adhesion of epidural space and multiple pathologies, because of the difficulty of correctly placing the various catheters at the target lesions [14,15,16,17]. Therefore, the long-term effects of percutaneous epidural adhesiolysis using these catheters are uncertain and controversial [8,11]. Previously, transforaminal balloon procedures have been shown to effect significant pain relief and improvement of functional outcomes in patients with chronic refractory lumbar foraminal stenosis [18]. Based on these considerations, a novel catheter (ZiNeu^®^, JUVENUI, Seongnam, Korea) was designed with an inflatable balloon attached to the end of the catheter tip [19]. In previous studies, the ZiNeu catheter was shown to be an effective alternative to other percutaneous epidural catheters in refractory spinal stenosis [20], and improvements were shown to be maintained for 12 months [21]. However, most elderly patients with degenerative spinal changes have multiple lesions. To our knowledge, the relationship between the successful adhesiolysis rate for multiple targets and improvement of clinical outcomes has not been investigated. 

In this study, we aimed to investigate the relationship of successful balloon adhesiolysis rate for multiple targets with clinical outcomes. Moreover, to maximize the generalizability of the findings and the possibility of external validation, patients in spine specialty hospitals as well as tertiary referral centers were included in the study.

## 2. Materials and Methods

This was a prospective, multicenter study conducted at the pain management clinics of five centers in the Republic of Korea (three spine specialty hospitals and two university-affiliated teaching hospitals). The study protocol was reviewed by the ethics committees or investigational review boards at each participating site. This study was registered in the Clinical Research Information Service in Republic of Korea (KCT 0002280).

### 2.1. Patients

Between July 2015 and April 2018, patients with chronic lumbar spinal stenosis, aged 20 years or older, suffering of intractable low back pain and/or lumbar radicular leg pain for more than 3 months were examined to ascertain their eligibility. A comprehensive assessment of medical history and physical examination were done on every patient to exclude a confounding disease as another cause of pain. To ascertain the diagnosis of spinal stenosis and determine the grade or level of spinal stenosis, lumbar magnetic resonance imaging (MRI) was performed on all patients. The degree of spinal stenosis was analyzed on the basis of MRI findings, as described in a previous study [22]. The inclusion criteria were as follows: chronic (more than 3 months) lumbar radicular pain, with or without low back pain; previously being refractory to conservative treatment; and failure of interlaminar epidural steroid injection or transforaminal epidural block (failed to maintain improvement for more than 1 month). Prior to enrollment, all eligible patients received a conventional diagnostic or therapeutic fluoroscopy-guided caudal or transforaminal epidural injection with local anesthetic and steroid. Patients with less than 50% pain improvement lasting less than one month following the epidural steroid injection were finally enrolled in this study. The exclusion criteria were as follows: patient refusal to participate in this study, age less than 20 years old, axial pain such as lumbar facet syndrome and myofascial pain syndrome, previous steroid injection within the previous 12 weeks, progressive neurological deficits or motor weakness, uncontrollable or unstable opioid use, previous side effects to steroids, coagulopathy, signs of infection, pregnancy or nursing, local anesthetics or contrast dye solutions, and unstable medical or psychiatric condition. 

### 2.2. Intervention: Percutaneous Epidural Decompression and Adhesiolysis Using an Inflatable Balloon Catheter

All procedures in the present study were done based in an outpatient setting. There were no sedatives or premedication administered prior to procedure. All procedures were performed under fluoroscopic guidance. The position of each patient was the prone position and the pillow was placed under the abdomen to minimize lumbar lordosis. After sterile preparation before the procedure, local anesthetic was infiltrated into the skin and soft tissue. A 10-gauge guide needle was specially designed for preventing the various types of potential damage of a catheter during catheter manipulation. The guide needle was gently introduced via the sacral hiatus under fluoroscopic guidance by experts with experience of more than 500 procedures. Consequently, about 8 mL of diluted contrast medium (Omnipaque, Nycomed Imaging AS, Oslo, Norway) was injected using the guide needle. The epidural space was confirmed by the spread of diluted contrast medium using fluoroscopy. The diluted contrast medium was prepared by mixing 4 mL of pure contrast medium, 4 mL of 1% lidocaine, and 1500 I.U. of hyaluronidase. By examining the contrast flow, filling defects or intravascular injections were identified. In the case of intravascular injection, the needle was repositioned. After suitable identification via an epidurogram of the target areas, a ZiNeu catheter was advanced via the guide needle to the filling defects or the sites of suspicious cause of pain, as determined based on both MRI findings and comprehensive assessment of symptoms before the procedure (Supplementary Table S1). At the planned target sites or filling defects (i.e., the central ventral and dorsal epidural space, the intervertebral disc area, the lateral recess area, right or left intervertebral foramen), mechanical adhesiolysis and balloon decompression were implemented using the ZiNeu catheter. Such epidural adhesiolysis and balloon decompression were performed via side-to-side positioning of the catheter with intermittent ballooning. The balloon of the catheter was prepared by filling 0.13 mL of contrast agent with a 1-mL Luer-Lock syringe (BD Medical, Franklin Lakes, NJ, USA), and every time ballooning was limited to 5 s [18]. For safety reasons, the time of balloon inflation was adjusted on the basis of the degree of pain caused by the procedure: if the patient complained of moderate to severe pain during balloon inflation, no further balloon adhesiolysis was attempted. The catheter moved only in the state of the balloon deflation. 

**Figure 1:**
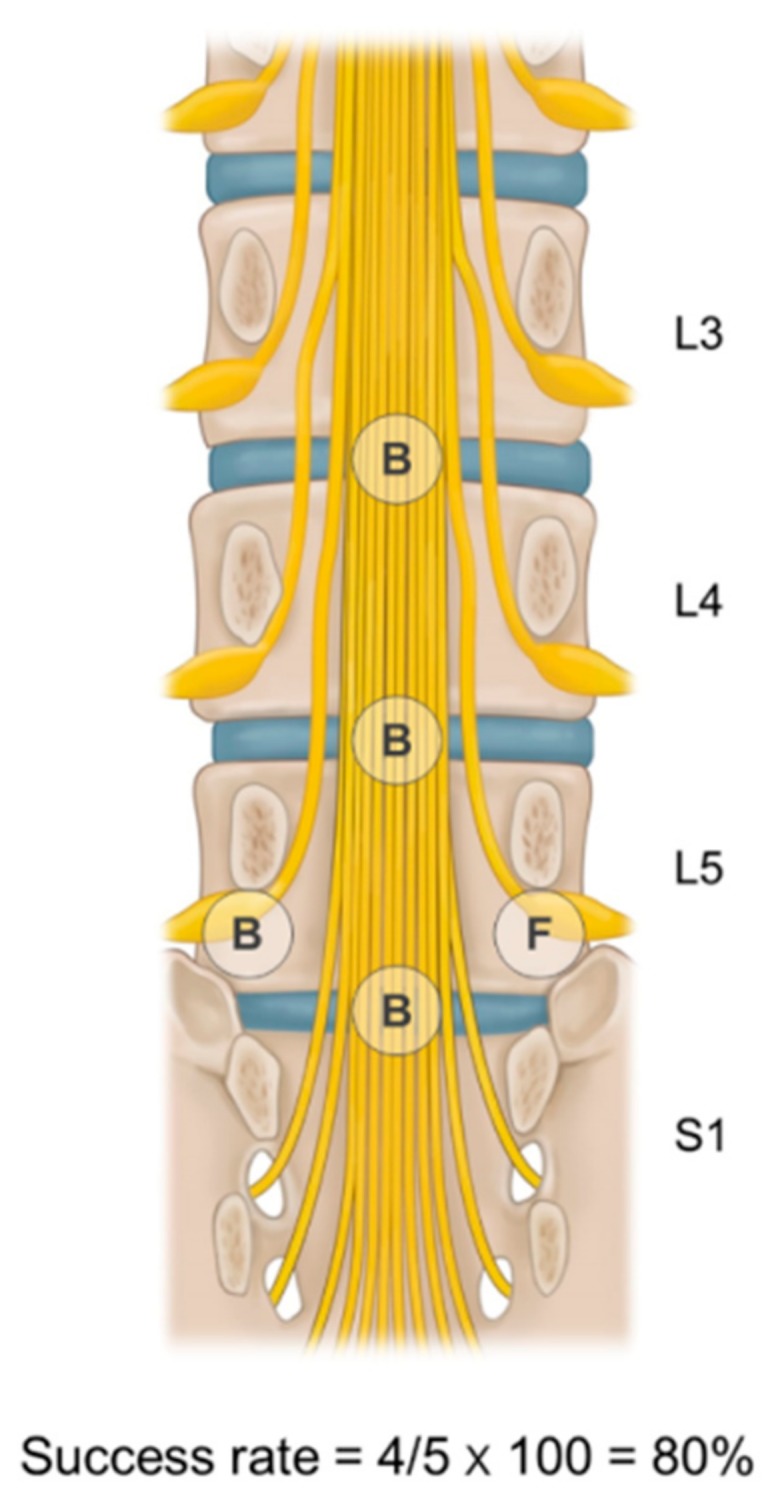
Determination of success rate of balloon adhesiolysis for multiple target sites. The rate of successful balloon adhesiolysis is defined as the number of successful ballooning procedures divided by the number of target lesions. In this schematic picture, the success rate is 80% (4 successful ballooning procedures out of 5 target lesions). B—ballooning success; F—failure of ballooning.

At every instance of epidural adhesiolysis and balloon decompression to each target site, 1 mL of contrast was injected to exclude subarachnoid or intravascular spread. Moreover, the ballooning process at each target site was recorded as successful if ballooning was adequately performed with enough dye spread, and as failed otherwise. The rate of successful balloon adhesiolysis was defined as the number of successful ballooning sites divided by the number of target lesions determined prior to balloon adhesiolysis (Figure 1). There were symptom-related multiple target sites in intractable stenosis in our study and the pain interventionists in our group attempted to perform epidural adhesiolysis at as many target sites as possible. Although epidural adhesiolysis for multiple target sites was not always conducted completely, we aimed to perform it on at least half of the interventional treatment targets. The mean balloon success rate in the present study was about 85%. Therefore, patients were categorized into one of three groups according to the balloon adhesiolysis success rate: above 85%, 50–85%, or below 50% of the total target sites. 

Then, 10 mL of 0.2% ropivacaine with 5 mg of dexamethasone was divided and injected separately at each target site. A Perifix epidural catheter (B. Braun Melsungen AG, Melsungen, Germany) was kept at the main lesion via the ZiNeu catheter lumen after the procedure had ended. In a recovery room, 2 mL of lidocaine was injected via the Perifix catheter to exclude the intrathecal injection. After 10 to 15 min of observation, 4 mL of 10% hypertonic saline was administered using the Perifix catheter. The patient was discharged from the outpatient surgery center with a Perifix catheter, and re-visited the outpatient clinic the following day. The same drug administered on the day of the procedure (10% hypertonic saline and steroid) was injected through a Perifix catheter on the second day of the procedure. After the drugs were administered, the catheter was removed. 

In case of suspected complications, such as a dura matter puncture, subdural injection, or vascular injection, the procedure was immediately stopped and recorded. Thereafter, the patient was transferred to a recovery room and a neurologic examination was performed. After bed rest for a short duration and confirmation of normal neurologic examination findings, the patient was discharged. 

### 2.3. Outcome Assessment and Follow-Up

The baseline characteristics of all study subjects were analyzed. Outcome assessments were conducted at baseline, and at 1, 3, and 6 months after the balloon adhesiolysis. Before the procedure was performed, all participants were instructed and evaluated on an 11-point NRS from 0 (no pain) to 10 (worst possible pain) to determine the intensity of both leg and lower back pain; the Korean version 10-item Oswestry disability index (ODI) questionnaire (range, 0–100; 0 = no disability) to determine physical functional status; and the Beck depression inventory to assess emotional status. The medication quantification scale III (MQS) was also measured to assess the changes in the usage of medication [23]. The global perceived effect (GPE) according to the 7-point Likert scale was also measured to analyze the patient’s satisfaction and improvement after the balloon adhesiolysis [24]. Side effects related to the procedure were also recorded. 

A multidimensional approach was used to analyze the study outcomes: the primary outcome was the number of patients with a successful response after balloon adhesiolysis at each follow-up. A successful responder was defined according to previous studies, with some modifications, as: 1) 50% (or ≥4 point) decrease of NRS from baseline, no increase from baseline ODI and MQS, and ≥4 points on the GPE scale; or 2) ≥30% (or ≥2-point) decrease of NRS from baseline together with any one of the following criteria: ≥30% (or ≥10 point) decrease in ODI from baseline, or ≥5 points on the GPE scale, or no increase from the baseline MQS. 

In addition, the NRS, ODI, MQS, and GPE scales of satisfaction were measured at 1, 3, and 6 after balloon adhesiolysis. The decreases in pain intensity, ODI, and MQS compared with baseline at each follow-up period were also recorded. Possible complications associated with the procedure were reported, and all side effects were further evaluated at follow-up visits. 

Patients were told to continue their previously prescribed analgesic medications if possible. Prior to participate in this study, all participants were told not to change any prescribed medications for the first month after the procedure, and they understood and agreed to these guidelines. The prescribed doses of each analgesic were then titrated based on the NRS at each follow-up period in the outpatient visit except opioids. Patients who needed to increase the dosage of analgesics, or who wanted other treatments, were regarded as treatment failures after the follow-up visit and recorded as having dropped out of the study. Patients who were lost to follow-up, prescribed an increased dose of opioid, performed other interventions, or were treated by surgery were also recorded to be treatment failures at that time-point. Each patient with treatment failure was defined as a non-responder at each follow-up visit.

### 2.4. Statistical Analysis

Categorical variables are expressed as numbers and percentages. Continuous variables are expressed as means with standard deviation (SD), 95% confidence intervals (CI), or medians with the interquartile range (IQR), as appropriate. To compare demographic data between the three groups, the chi-square test or Fisher exact test was used to assess categorical data, as appropriate. One-way ANOVA or Kruskal–Wallis tests were used to analyze numerical data, as appropriate. All data were measured and analyzed on an intent-to-treat basis, regardless of follow-up loss or withdrawal from the study. Because of data loss resulting from drop-outs, a linear mixed effect model (LMEM) was used to compare changes within and between groups in terms of continuous variables (NRS, ODI, MQS, and GPE) at baseline and 1, 3, and 6 months after balloon adhesiolysis. To compare repeated data of successful responders (binary outcome) among groups, a generalized estimating equation was used. Data were analyzed using the Statistical Package for the Social Sciences (SPSS version 21.0, SPSS Inc., Chicago, IL, USA) or SAS version 9.3 (SAS institute, Cary, NC, USA). A two-tailed *p*-value < 0.05 was considered to indicate a statistically significant difference.

## 3. Results

### 3.1. Demographics

**Figure 2:**
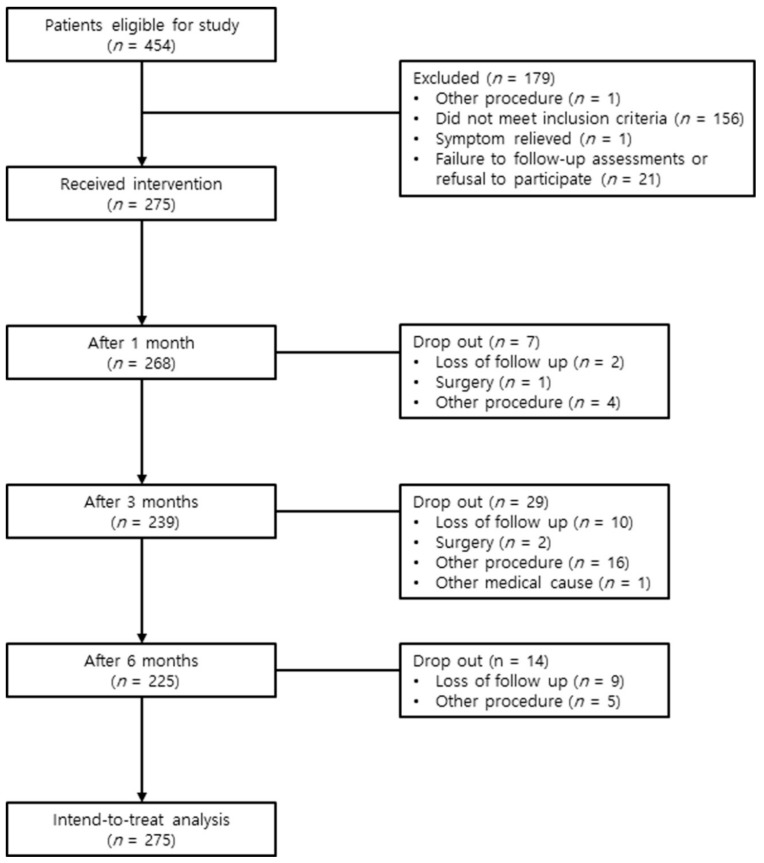
Study flow diagram.

A series of 454 patients who had been diagnosed with lumbar spinal stenosis between July 2015 and April 2018 were screened for eligibility to participate in this study. These patients presented with chronic lumbar radicular pain, with or without lower back pain. Among these patients, 156 did not meet inclusion criteria, 21 declined to participate in the study or did not visit again, 1 received another procedure, and 1 experienced relief from the symptoms. Ultimately, 275 patients received the intervention (Figure 2). The baseline patient demographic characteristics are shown in Supplementary Table S1.

### 3.2. Ballooning Success Rate Groups for Multiple Target Sites

Among these patients, 48, 79, and 148 patients were included in 50%, 50–85%, and above 85% ballooning success rate groups for multiple target sites, respectively. The baseline characteristics and intervention characteristics of patients of the three groups are presented in Table 1 and Table 2, respectively. Compared with the patients in the above 85% and 50–85% success groups, those in the below 50% success group were older (61.6 (13.1) vs. 61.9 (12.8) vs. 68.9 (12.2), respectively, *p* = 0.003). Compared with the patients in the above 85% and 50–85% success groups, those in the below 50% success group had lower body mass index (24.2 (2.9) vs. 24.8 (2.9) vs. 23.2 (4.1), respectively, *p* = 0.019). There was no significant difference in demographic data other than age and body mass index between the three groups (Table 1 and Table 2). 

### 3.3. The Estimated Proportions of Successful Responders

The estimated proportions of successful responders in the below 50% group at 1, 3, and 6 months after procedure were 0.688, 0.542, and 0.292, respectively. For the 50–85% group, the respective proportions were 0.633, 0.582, and 0.468. Lastly, in the above 85% success rate group, the estimated proportions at 1, 3, and 6 months after the procedure were 0.662, 0.628, and 0.507, respectively. The group difference was not statistically significant; however, the time difference was statistically significant (p = 0.358 and p < 0.001, respectively). The p-value of the interaction between groups and time for successful responders was 0.096 (Table 3). The observed numbers of patients who satisfied the individual criteria for successful response at each follow-up visit are shown in Supplementary Table S2.

### 3.4. The Estimated Mean Changes in the Back and Leg Pain, and ODI

**Figure 3:**
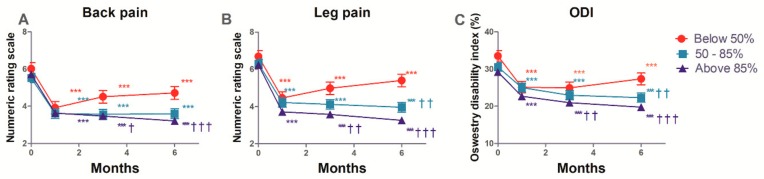
Numerical rating scale (NRS) of back (**A**) and leg (**B**) pain, and Oswestry disability index (ODI; (**C**)) at baseline (0), and at 1, 3, and 6 months after epidural decompression and adhesiolysis with a balloon-inflatable catheter. The patients were divided into groups of less than 50%, 50–85%, and 85% success rate depending on the rate of successful ballooning procedures per target site. ** *p* < 0.01 vs. baseline. *** *p* < 0.001 vs. baseline. ^†^ *p* < 0.05 compared with the below 50% group at each time point. ^††^ *p* < 0.01 compared with the below 50% group at each time point. ^†††^ *p* < 0.001 compared with the below 50% group at each time point. The data are presented as estimated mean ± 95% confidence interval.

The estimated mean changes from baseline in the NRS of back and leg pain, and ODI functional status over the 6 months of follow-up, are shown in Supplementary Table S3 and Figure 3. These intent-to-treat analyses show that after balloon adhesiolysis with a ZiNeu catheter, pain intensity (both in the lower back and the legs), and functional capacity based on ODI, were significantly improved compared with baseline in all three groups, and the improvements were maintained during the 6 months follow-up period. Concerning back pain, there was a significant difference at 3 and 6 months in both 50–85% and above 85% success rate groups compared with the below 50% group. The same significant differences were observed in leg pain. Similarly, significant differences in functional capacity were observed at 6 months in the 50–85% group. As shown in Table 4, the time effect for GPES was statistically significant (*p* < 0.001), and the group effect for GPES was not (*p* = 0.346). The interaction between group and time for GPES was statistically significant (*p* = 0.010). 

### 3.5. Observed Complications

Cumulative lists and rates of the observed complications during the decompression and adhesiolysis with an inflatable balloon catheter are shown in Table 5. Suspicious dura puncture was found in 3.15% of all patients, and subdural injection was suspected in 1.58% of the cases. Moreover, the prevalence of vascular injection was 1.26%, and 1.89% of the patients showed hypotension during balloon adhesiolysis. However, none of the patients experiencing complications had persistent neurologic abnormalities, and all were discharged after bed rest for a short duration.

## 4. Discussion

In the present study, we analyzed the relationship of successful ballooning rate for total target sites with clinical outcomes such as back and leg pain, and functional capacity based on ODI. Our study suggests that the more successful balloon adhesiolysis procedures for multiple target lesions that are performed, the better the clinical outcome that may be expected at least 6 months after treatment. 

In contrast with the presence of a single lesion (e.g., a single level intervertebral disc herniation in young patients), the presence of many potential lesions, as observed in elderly patients with degenerative spinal disease, is often associated with pain or functional disability [7,21,25]. In our patients, the number of target levels varied between 1 and 4 (Table 1). Because of the difficult approach to the lesions, anatomic variation, or the level of experience of the physician, epidural adhesiolysis to all target lesions is difficult in some cases, and partial adhesiolysis is not uncommon. In this multicenter prospective study, the success rate of balloon adhesiolysis for multiple target sites was also variable. For reasons similar to the case of other epidural adhesiolysis, balloon procedure to all target lesions was also difficult in some cases. Moreover, balloon adhesiolysis to all target lesions might be more difficult than simple epidural adhesiolysis without a balloon. In some cases, balloon adhesiolysis could not be performed, and simple epidural adhesiolysis was performed instead. In this study, such cases were recorded as ballooning failures. To our knowledge, there have been few reports about the clinical outcome of partial adhesiolysis compared to adhesiolysis of all target lesions. More importantly, this is the first study of the relationship between the successful rate of balloon adhesiolysis for multiple target lesions and clinical outcomes. Compared with the below 50% balloon success rate group, the 50–85% and above 85% success rate groups were characterized by greater effectiveness for at least 6 months. In other words, the better balloon adhesiolysis is performed, the better the clinical outcome that can be expected. However even the below 50% group showed significant benefits lasting 6 months, even if the effects in this group were less pronounced than those in the other groups. Moreover, since this is a multicenter study, our findings may be more generalizable than those of a single center study [26,27]. 

The efficacy of percutaneous epidural adhesiolysis in chronic low back pain refractory to conservative therapies has been relatively well investigated [8,11]. Various catheters such as shearing-resistant catheters (the Racz-type catheter), or steerable navigation catheters (e.g., NaviCath, Myelotec, Roswell, GA, USA), designed to deliver medication to stenotic or adhesion sites, have been reported to be clinically effective in treating intractable chronic pain [13,14,16]. However, epidural adhesiolysis using these catheters has some limitations in patients with severe stenosis or adhesion because of the difficulty in handling and navigating the catheter tip to the target lesion [8,9,16,28]. On the other hand, epidural adhesiolysis and balloon decompression using a balloon inflatable catheter might be effective when other percutaneous epidural catheters fail to remove the adhesions or to sufficiently improve the clinical outcome [20,21]. The significant pain relief and functional improvement induced via balloon adhesiolysis using the ZiNeu catheter was maintained for 12 months, especially in terms of the ODI [20,21]. This effect in refractory spinal stenosis seems to be possible because more epidural medication was delivered by direct balloon dilation of the stenotic lesion than by using other catheters. 

This study has several potential limitations. The definition of successful responder could conceivably be criticized, as different results might have been obtained if the definition had been changed. Therefore, the definition of successful responder was determined according to previous reports and recommendations [29,30,31,32]. We carefully chose our definition of response to reflect treatment success combined with patient-reported outcomes, including ODI, GPE, and MQS [32,33,34]. Moreover, loss to follow-up, withdrawal from the study, or the performance of other procedures or surgery was considered as treatment failure. The dropout rate for the 6 months follow-up was 21.8%. To control for this effect, we used the LMEM for statistical analysis. Compared with analysis of variance, LMEM is known to be more flexible in accommodating longitudinal data features, and can more efficiently achieve greater power in datasets with missing data [32,35]. In addition, the balloon decompression and epidural adhesiolysis considered in this study was a complex treatment consisting of several components, such as irrigation with normal saline, ballooning, administration of various drugs, and administration of hypertonic saline 2 days after the procedure. Therefore, we could not rule out the possibility that other components of this treatment have provided the essential therapeutic effect. However, all patients in this study received the same procedure, except for the success rate of balloon adhesiolysis for multiple target sites. Furthermore, the proportion of successful responders or other clinical outcomes can be, at least in part, influenced by various confounding factors. Since there was no control group in our study, a randomized controlled trial may be needed to address this limitation. Therefore, we plan to perform a further randomized control study of balloon adhesiolysis. Lastly, the heterogeneity in clinical practice among the various centers in this multicenter study may be a confounding factor in interpreting the results of the study [26]. Therefore, we made an effort to reduce the discrepancy by implementing and strictly monitoring rigorous study protocols and inclusion/exclusion criteria [26,36]. 

## 5. Conclusions

The balloon decompression and epidural adhesiolysis with a ZiNeu catheter can lead to significant pain relief and functional improvement lasting at least 6 months in patients with chronic refractory spinal stenosis. Compared with balloon success rates below 50% for multiple target sites, success rates greater than 50% may lead to more significant improvements in back pain, leg pain, and functional capacity for at least 6 months.

## Figures and Tables

**Table 1:** Baseline characteristics of the study subjects according to the success rate of balloon adhesiolysis for multiple targets.

Parameter	Below 50% (*n* = 48)	50–85% (*n* = 79)	85–100% (*n* = 148)	*p*-Value
Age, years	68.9 (12.2)	61.9 (12.8)	61.6 (13.1)	0.003
Gender, *n* (%)				
Male/female	22/26 (45.8/54.2)	48/31 (60.8/39.2)	79/69 (53.4/46.6)	0.251
Body mass index, kg/m^2^	23.2 (4.1)	24.8 (2.9)	24.2 (2.9)	0.019
Areas of pain, *n* (%)				0.943
Back/leg/both	7/4/37 (14.6/8.3/77.1)	11/8/60 (13.9/10.1/76.0)	17/17/114 (11.5/11.5/77.0)	
Duration of pain (months)	12.0 (4.0–24.0)	12.0 (7.0–36.0)	12.0 (6.0–24.0)	0.771
Concurrent disease, *n* (%)				
Diabetes	10 (20.8)	8 (10.1)	27 (18.2)	0.189
Hypertension	30 (62.5)	38 (48.1)	74 (50.0)	0.244
Cardiovascular disease	14 (29.2)	35 (44.3)	43 (29.1)	0.053
Spinal stenosis grading, *n* (%)				
Central canal (A/B/C/D)	9/11/9/1 (18.8/22.9/18.8/2.1)	16/15/23/0 (20.3/19.0/29.1/0.0)	35/20/25/3 (23.6/13.5/16.9/2.0)	0.355
Foraminal (mild/moderate/severe)	11/17/20 (22.9/35.4/41.7)	28/22/24 (35.4/27.8/30.4)	51/33/34 (34.5/22.3/23.0)	0.190
Spondylolisthesis, *n* (%)	6 (12.5)	5 (6.3)	13 (8.8)	0.480
MQS, points	11.8 (8.0–15.0)	11.0 (8.0–12.2)	8.0 (7.6–11.2)	0.100
Pain intensity (NRS)				
Back	7.0 (4.0–8.0)	6.0 (4.0–7.0)	6.0 (4.0–8.0)	0.414
Leg	7.0 (5.5–8.0)	6.0 (5.0–8.0)	7.0 (5.0–8.0)	0.515
ODI (%)	34.0 (24.5–39.5)	30.0 (23.0–38.0)	28.0 (22.0–35.0)	0.071
BDI, points	7.0 (5.0–10.0)	6.0 (5.0–9.0)	6.0 (4.0–11.0)	0.595

The patients were divided into groups of less than 50%, 50–85%, and 85–100% depending on the success rate of the ballooning procedure for multiple target sites. Data are expressed numbers (%), and means ± standard deviation, or medians (interquartile range). MQS = medication quantification scale; NRS = numeric rating scale; ODI = Oswestry disability index; BDI = Beck depression inventory.

**Table 2:** Intervention characteristics of the study subjects according to the success rate of balloon adhesiolysis for multiple targets.

Parameter	Below 50% (*n* = 48)	50–85% (*n* = 79)	85–100% (*n* = 148)	*p*-Value
Target level, *n* (%)				0.284
1 level (L3-4/L4-5/L5-S1) 2 levels (L3-4-5/L4-5-S1) 3 levels (L2-3-4-5/L3-4-5-S1) 4 levels (L2-3-4-5-S1)	0/24/3 (0.0/50.0/6.3) 5/15 (10.4/31.3) 0/1 (0.0/2.1) 0 (0.0)	2/31/10 (2.5/39.2/12.7) 9/24 (11.4/30.4) 0/3 (0.0/3.8) 0 (0.0)	2/56/9 (1.4/37.8/6.1) 21/40 (14.2/27.0) 5/12 (3.4/8.1) 3 (2.0)	
Target site, *n* (%)				0.302
Left/right/both/central/Lt, central/Rt, central/both, central	8/6/14/1/4/5/ 10 (16.7/12.5/29.2/2.1/8.3/10.4/20.8)	13/6/24/2/4/7/23 (16.5/7.6/30.4/2.5/5.1/8.9/29.1)	19/13/40/17/17/12/30 (12.8/8.8/27.0/11.5/11.5/8.1/20.3)	
Number of target sites, *n* (%)				0.368
2–3/4–5/above 6	18/22/8 (37.5/45.8/16.7)	21/35/23 (26.6/44.3/29.1)	54/57/37 (36.5/38.5/25.0)	

The patients were divided into groups of less than 50%, 50–85%, and 85– 00% depending on the success rate of the ballooning procedure for multiple target sites. Data are expressed numbers (%). Lt, left; Rt, right.

**Table 3:** Estimated proportions of successful responders among patients who were treated by the decompression and adhesiolysis using an inflatable balloon catheter.

	Follow-Up (Months)	Below 50% (*n* = 48), Estimated Proportion (95% CI)	50–85% (*n* = 79), Estimated Proportion (95% CI)	85–100% (*n* = 148), Estimated Proportion (95% CI)	*p*- Value
Successful	1	0.688 (0.556–0.819)	0.633 (0.527–0.739)	0.662 (0.586–0.738)	0.812
Responder	3	0.542 (0.401–0.683)	0.582 (0.474–0.691)	0.628 (0.551–0.706)	0.528
	6	0.292 (0.163–0.420)	0.468 (0.358–0.578)	0.507 (0.426–0.587)	0.038

The patients were divided into groups of less than 50%, 50–85%, and 85–100% depending on the success rate of the ballooning procedure for multiple target sites. Successful response was defined as: 1) ≥50% (or ≥4-point) reduction from baseline leg NRS; and no increase from baseline ODI and MQS; and ≥4 points on the GPES scale; or 2) ≥30% (or ≥2-point) reduction from baseline NRS with any one of the following criteria; simultaneous ≥30% (or ≥10-point) reduction in ODI from baseline; or ≥5 points on the GPE scale; or no increase from the baseline MQS. A generalized estimating equation was used in the statistical analysis. Data are expressed as estimated proportions and 95% confidence interval (CI). GPES = global perceived effect of satisfaction; MQS = medication quantification scale; ODI = Oswestry disability index.

**Table 4:** Changes in the estimated global perceived effect of satisfaction in patients who were treated using decompression and adhesiolysis using an inflatable balloon catheter.

Variables *	Time (Months)	Below 50% (*n* = 48) Values (95% CI)	50–85% (*n* = 79) Values (95% CI)	85–100% (*n* = 148) Values (95% CI)	*p*-Value ^†^
GPES	1	4.80 (4.43–5.17)	4.83 (4.55–5.11)	4.86 (4.64–5.09)	0.955
	3	4.86 (4.05–5.67)	4.23 (3.47–4.99)	4.48 (3.98–4.99)	0.532
	6	4.09 (3.68–4.49)	4.31 (4.01–4.61)	4.88 (4.64–5.12)	0.001

The patients were divided into groups of less than 50%, 50–85%, and 85–100% depending on the success rate of the ballooning procedure for multiple target sites. * GPES was measured after decompression and adhesiolysis with an inflatable balloon catheter. ^†^ A linear mixed model was used in the statistical analysis. The group difference was not significant (*p* = 0.346). The time effect was significant (*p* < 0.001). The *p*-values of interaction between the group and time was significant (*p* = 0.010). CI = confidence interval. GPES = global perceived effect of satisfaction.

**Table 5:** Cumulative list and rate of the observed complications during the decompression and adhesiolysis with an inflatable balloon catheter.

Complication	Number (%)
Dura matter puncture	9 (3.3)
Subdural injection	5 (1.8)
Vascular injection	4 (1.5)
Disc injection	6 (2.2)
Hypotension	4 (1.5)

## Supplementary Materials

The following are available online at https://www.mdpi.com/2077-0383/8/5/606/s1. Table S1: Baseline characteristics of the study subjects. Table S2: Observed number of patients who satisfied the individual parameters of successful response at each follow-up visit. Table S3: Changes in the estimated mean pain score and physical function in patients who were treated by decompression and adhesiolysis using an inflatable balloon catheter.
