# Supplementary material for: Relationship of Success Rate for Balloon Adhesiolysis with Clinical Outcomes in Chronic Intractable Lumbar Radicular Pain: A Multicenter Prospective Study
Source: J Clin Med. 2019 May 3;8(5):606. doi: 10.3390/jcm8050606 (PMC6572522; doi:10.3390/jcm8050606)
Supplement: Supplementary file 1 [file jcm-08-00606-s001.pdf]

Supplemental Table S1. Baseline characteristics of the study subjects.

**Parameter**	***n* = 275**
Age, years	62.0 (13.8)
Sex, *n* (%)	
Male/female	149/126 (54.2/45.8)
Body mass index, kg/m^2	24.2 (3.2)
Areas of pain, *n* (%)	
Back/leg/both	35/29/211 (12.7/10.5/76.7)
Duration of pain (months)	12.0 (7.0–24.0)
Concurrent disease, *n* (%)	
Diabetes	45 (16.4)
Hypertension	160 (58.2)
Cardiovascular disease	92 (33.5)
Spinal stenosis grading, *n* (%)	
Central canal (A/B/C/D)	60/46/57/4 (21.8/16.7/20.7/1.5)
Foraminal (mild/moderate/severe)	90/72/78 (32.7/26.2/28.4)
Spondylolisthesis, *n* (%)	24 (8.7)
Target level, *n* (%)	
1 level (L3-4/L4-5/L5-S1)	4/111/22 (1.5/40.4/8.0)
2 levels (L3-4-5/L4-5-S1)	35/79 (12.7/28.7)
3 levels (L2-3-4-5/L3-4-5-S1)	5/16 (1.8/5.8)
4 levels (L2-3-4-5-S1)	3 (1.1)
Target site, *n* (%)	
Left/right/both/central	40/25/78/20 (14.6/9.1/28.4/7.3)
Left, central/right, central/both, central	25/24/63 (9.1/8.7/22.9)
Number of target site, *n* (%)	
2–3/4–5/above 6	93/114/68 (33.8/41.5/24.7)
Success rate of ballooning for targets, *n* (%)	
0-50%/50-85%/85-100%	48/79/148 (17.5/28.7/53.8)
Medication quantification scale, points	8.8 (8.0–12.4)
Pain intensity (numeric rating scale)	
Back/Leg	6.0 (4.0–8.0)/7.0 (5.0–8.0)
Oswestry Disability Index (%)	30.0 (22.5–36.5)
Beck depression inventory	6.0 (4.0–9.0)

Data are expressed as numbers (%), or mean ± standard deviation, or medians (interquartile range).

Supplemental Table S2. Observed number of patients who satisfied the individual parameters of successful response at each follow-up visit.

Parameters	Follow-Up (Months)	Below 50% (*n* = 48) Number (%)	50–85% (*n* = 79) Number (%)	85–100% (*n* = 148) Number (%)
≥50% (or ≥4-point) reduction in NRS	1	20 (41.7)	36 (45.6)	70 (47.3)
	3	11 (22.9)	38 (48.1)	68 (45.9)
	6	12 (25.0)	38 (48.1)	75 (50.7)
≥30% (or ≥2-point) reduction in NRS	1	33 (68.8)	53 (67.1)	101 (68.2)
	3	27 (56.3)	51 (64.6)	96 (64.9)
	6	22 (45.8)	48 (60.8)	90 (60.8)
≥30% (or ≥10-point) reduction in ODI	1	18 (37.5)	19 (24.1)	53 (35.8)
	3	19 (39.6)	28 (35.4)	62 (41.9)
	6	13 (27.1)	28 (35.4)	48 (32.4)
No change or reduction in MQS	1	37 (77.1)	59 (74.7)	98 (66.2)
	3	41 (74.55)	54 (62.07)	113 (64.57)
	6	5 (10.4)	5 (6.3)	9 (6.1)
≥5 points in GPES	1	29 (60.4)	46 (58.2)	81 (54.7)
	3	27 (56.3)	46 (58.2)	89 (60.1)
	6	10 (20.8)	25 (31.6)	66 (44.6)

The patients were divided into three groups—less than 50%, 50–85%, and 85–100%—depending on the success rate of the ballooning procedure for multiple target sites. Data are expressed as numbers (%). NRS, numerical rating scale; ODI, Oswestry disability index; MQS, medication quantification scale; and GPES, global perceived effect of satisfaction.

Supplemental Table S3. Changes in the estimated mean pain score and physical function in patients who were treated using decompression and adhesiolysis using an inflatable balloon catheter.

Variables *	Time (Months)	Below 50% (n = 48) Values (95% CI)	50–85% (n = 79) Values (95% CI)	85–100% (n = 148) Values (95% CI)	p-Value †
Back pain	Baseline	6.02 (5.39–6.66)	5.52 (5.02–6.01)	5.72 (5.36–6.08)	0.473
	1	3.92 (3.27–4.57)	3.61 (3.10–4.12)	3.63 (3.26–4.00)	0.717
	3	4.51 (3.84–5.18)	3.57 (3.05–4.09)	3.47 (3.09–3.85)	0.026
	6	4.72 (4.04–5.40)	3.60 (3.07–4.12)	3.21 (2.82–3.61)	0.001
Leg pain	Baseline	6.69 (6.08–7.30)	6.30 (5.83–6.78)	6.24 (5.89–6.59)	0.454
	1	4.46 (3.83–5.09)	4.22 (3.72–4.71)	3.71 (3.36–4.07)	0.073
	3	4.98 (4.33–5.63)	4.12 (3.61–4.62)	3.58 (3.21–3.94)	0.001
	6	5.40 (4.73–6.06)	3.96 (3.45–4.48)	3.26 (2.88–3.64)	<0.001
ODI	Baseline	33.54 (30.76–36.32)	30.57 (28.37–32.77)	29.21 (27.50–30.91)	0.034
	1	25.12 (22.17–28.07)	25.00 (22.69–27.31)	22.68 (20.91–24.45)	0.188
	3	24.94 (21.90–27.99)	22.97 (20.59–25.36)	20.95 (19.11–22.79)	0.072
	6	27.37 (24.27–30.46)	22.32 (19.90–24.74)	19.76 (17.87–21.65)	<0.001

The patients were divided into three groups—less than 50%, 50–85%, and 85–100%—depending on the success rate of the ballooning procedure for multiple target sites. A numerical rating scale was used to assess the intensity of both lower back and leg pain. Oswestry disability index (ODI) was used to assess physical function. * Outcome variables measured after decompression and adhesiolysis with an inflatable balloon catheter. † A linear mixed model was used in the statistical analysis. Omnibus p of back pain, leg pain, and ODI were 0.007, 0.001, and 0.087, respectively. CI = confidence interval.
